# One Health strategies in combating antimicrobial resistance: a Southeast Asian perspective

**DOI:** 10.7189/jogh.15.03025

**Published:** 2025-06-06

**Authors:** Pooi Yin Chung

**Affiliations:** Department of Pathology and Pharmacology, School of Medicine, IMU University, Kuala Lumpur, Malaysia

## Abstract

Antimicrobial resistance (AMR) is a multifactorial global public health concern that is interlinked with the health of humans, animals, and the environment. Therapeutically important antibiotics used widely in the mass medication of livestock have contributed significantly to AMR, as they eventually enter the environment due to inadequate treatment of wastewater. Similarly, improper discharge of antibiotic-contaminated waste by the medical sector exacerbates the problem by contributing to the increase in the selection of resistant bacteria and the horizontal transfer of resistance genes. Developed and developing countries and regions worldwide acknowledged that no discipline or sector of society can successfully address climate change, known and emerging infectious diseases, and AMR by acting in isolation. Countries in Southeast Asia, like elsewhere in the world, have also adopted a transdisciplinary and multi-sectoral collaboration integrating human, animal, and environmental health, known as the One Health strategy.

Antimicrobial resistance (AMR) is a significant and growing public health concern worldwide, and Southeast Asia is no exception. The region’s demand for animal-source proteins, particularly poultry and pork, has increased significantly in the past five decades, and the related intensification of livestock production has been accompanied by an increase in antimicrobial consumption and, consequently, AMR. Over the past two decades, critically important antimicrobials such as beta-lactams, aminoglycosides, macrolides, and quinolones have been administered for long periods to promote growth and improve feed efficiency [1]. In Malaysia, *Salmonella* species isolated from chickens showed increased resistance to all antimicrobials, notably chloramphenicol, ciprofloxacin, tetracycline, and colistin. Antimicrobials are also used prophylactically and therapeutically to control disease outbreaks in aquaculture [2]. In Southeast Asian countries, *Escherichia* *coli*, *Aeromonas* sp and *Vibrio* sp are the most widely and frequently reported multidrug-resistant bacteria in aquaculture [3,4].

In public health surveillance on resistance patterns in hospitals in Malaysia, methicillin-resistant *Staphylococcus aureus*, *Staphylococcus pneumoniae*, *Enterococcus faecalis*, *E*. *faecium*, *Acinetobacter baumannii*, *Pseudomonas aeruginosa*, *K*. *pneumoniae*, and *E*. *coli* showed resistance to all tested antibiotics [5]. In the community setting, *E coli* in patients with urinary tract infections showed the highest resistance rate to ampicillin, among other antibiotics [5]. Similar resistance patterns were noted in Thailand, Indonesia, Vietnam and Singapore [6–9].

In Southeast Asia, wildlife is indiscriminately hunted, consumed, and traded for food or use in traditional medicine, which increases the risk of pathogen spillover between humans and animals. The growth and expansion of human populations into new geographical areas has an impact similar to that of global warming and climate change, resulting in the shift in the migration patterns of wildlife.

Re-emerging zoonotic diseases of particular significance in Southeast Asia include avian influenza, rabies, and leptospirosis, among others. From 2003 to 2024, 889 cases of avian influenza were reported in 23 countries worldwide with 52% fatality rate [10]. During the past three decades, Malaysia experienced four waves of H5N1 highly pathogenic avian influenza outbreaks [11]. In 2005–17, Indonesia detected and reported 200 H5N1 infections in humans, of which 168 (84%) were fatal [10]. Although it recorded no human infections since 2020, Indonesia still has the second-highest reported cases and the highest reported case-fatality proportion among all countries reporting H5N1 infections in humans. In Vietnam, 127 human infections with H5N1, including 64 deaths, have been reported from 2003 to 2014. The most recent H5N1 fatality was reported in Mar 2024 from the Northern Province of Phu Tho, Vietnam [12]. There are currently no known cases of avian flu in Singapore, mainly due to its strict biosecurity measures and surveillance [13].

Rabies is included in the World Health Organization (WHO) 2021–2030 Roadmap for Neglected Tropical Diseases, which sets regional, progressive targets for the global strategic plan to end human deaths from dog-mediated rabies by 2030 [14]. There are an estimated 59 000 deaths from rabies globally each year. Malaysia recorded 72 fatalities since the outbreak was declared in July 2017, including four fatalities between January and June 2024 [15]. Rabies is endemic in 26 provinces in Indonesia, with 74 human cases reported from January to July 2023 [16]. It remains an unresolved public health problem in Thailand and Vietnam [17,18] while Singapore has been rabies-free since 1953 [19].

Leptospirosis, another zoonotic disease, spreads via contact with the urine of infected rodents. At least 33 000 cases with 450 deaths have been recorded in Malaysia since its classification as a notifiable disease in 2010 [20]. In Indonesia, 920 leptospirosis cases were reported, with 12 deaths [21], while Thailand reported a case fatality rate of 0.04 cases per 100 000 population between 2020 and 2022 [22].

Southeast Asia has been experiencing warmer climates and record-breaking temperatures in recent years. Consequent increases in precipitation have led to increased enrichment with nutrients, particularly nitrogen and phosphorus from agricultural runoff, wastewater discharge, and industrial pollution (eutrophication), and rapid and excessive increase of bacterial populations (bacterial blooms) in water systems. The heightened concentration of bacteria creates a greater risk for the transfer of antibiotic-resistance genes [23]. At the same time, rapidly industrialising countries such as Malaysia, Indonesia, and Vietnam are particularly impacted by microplastic pollution in water sources, which increases the dissemination of antibiotic-resistant genes in bacteria [24].

The WHO, in collaboration with the Food and Agriculture Organization (FAO) and World Organization for Animal Health (WOAH, founded as the *Office International des Epizooties*), launched the Global Action Plan (GAP) against AMR in 2015 to ensure treatment and prevention of infectious diseases are achievable with quality-assured, safe, and effective medicines [25]. In response to the GAP, member states from Southeast Asia have developed their context-specific national action plans on AMR based on the One Health approach to address specific challenges and priorities. One Health is a collaborative and multidisciplinary approach that emphasises the interconnectedness of human, animal, and environmental health in achieving optimal health outcomes.

Malaysia developed the action plans on AMR for 2017–2021 and 2022–2026 which focus on public awareness and education, surveillance and research, infection prevention and control, and appropriate use of antimicrobials in health care facilities, animal health sectors, non-livestock agriculture, and environment sectors. Singapore has a similar framework for strengthening and enhancing activities to combat AMR, addressing identified gaps, and prioritising future interventions [5]. Surveillance systems and programmes such as the Communicable Diseases Live & Enhanced Surveillance system, the Acute Respiratory Infection Surveillance Program, and the biosurveillance system have been established to support the One Health strategy in enabling timely disease notifications and updates [26]. Academic institutions and research centres in Singapore support the One Health strategy through the Programme for Research in Epidemic Preparedness and Response to enhance research and innovation in epidemic preparedness [27].

In Thailand, a raised-without-antibiotics initiative was implemented in swine farms to regulate the production of medicated feeds with antibiotics [28]. Fishery biologists, aquatic animal health professionals and veterinarians have jointly developed standards for animal disease control and prevention, and appropriate use of antimicrobials in aquaculture [29]. Indonesia has developed *Sistem Informasi Zoonosis dan Emerging Infectious Diseases*, a One Health information-sharing platform, to facilitate rapid response to these diseases [30]. In Vietnam, the use of antibiotics as growth promoters in livestock has been banned since 2018, while from 2020 onwards, all antibiotic use in animals has required a prescription, with prophylactic use in livestock to be phased out by 2026 [31]. Proper husbandry and animal welfare practices, and the efficient and relevant use of vaccines to minimise the use of antibiotics have also been planned [31].

Despite the growing recognition of the One Health approach in combating the AMR crisis, its implementation poses challenges related to policy implementation and enforcement, financial and human resources, surveillance systems for standardised data collection and reporting, and public awareness. In Malaysia, monitoring and enforcement are poor although policies and acts to regulate antibiotic use in health care and animal feed are in place [32]. In Thailand, a comprehensive surveillance system and data integration platform to monitor the prevalence and relationship of targeted bacteria across the human, livestock, aquaculture, food, and environmental sectors has yet to be fully developed and implemented. Furthermore, the prescription and dispensing of antimicrobials for animals are neither legally regulated nor systematically audited [33].

In Indonesia, antibiotic prescription practices in health care facilities and communities are poor overall, and there is a substantial lack of AMR awareness and knowledge about antibiotics among the poor and lower-educated [34]. Self-medication with antibiotics without proper prescription is also common, as is their dispensing over-the-counter in pharmacies across the country [34]. Despite guidelines on antibiotic use, updated diagnostic and treatment, and classification of antibiotic use in hospital databases in Vietnam, inappropriate antibiotic use persists in hospitals [35]. Singapore, a well-resourced country, has well-functioning stewardship programmes, comprehensive hospital surveillance systems, and the latest technologies and innovations to address AMR. However, surveillance in private clinics, resources at the community level, and public awareness and regulation on antimicrobial use in animals remain poor. Farmers in the agriculture and aquaculture sectors do not have to obtain prescriptions to access antibiotics. The vested interests of pharmaceutical companies and hospitals in antibiotic prescription hinder the introduction of stewardship programmes to curb antibiotic prescription. Additionally, the public lacks proper understanding of antibiotics and has limited knowledge about their appropriate use [36].

In recognition of the fact that zoonotic diseases contribute significantly to the emergence and spread of AMR, and the importance of a multi-sectoral and collaborative One Health approach between countries and between governmental and non-governmental entities in the region in tackling the threat of AMR, various initiatives have been established [37,38]. The Southeast Asian One Health Universities Network was set up in 2011 to improve One Health workforce capacity by leveraging education and training to equip the next generation of professionals with problem-solving skills that address the interconnected human, animal, and ecosystem health. Similarly, the Association of Southeast Asian Nations (ASEAN) set up several initiatives, as follows:

- the ASEAN Coordinating Centre for Animal Health and Zoonosis, founded in 2016 to facilitate cooperation among ASEAN member states, speed up regional coordination for the prevention and control of zoonotic diseases and enable collaboration with public health and environment sectors to promote global health, food safety, and food security:
- the ASEAN Strategy for Exotic, Emerging, Re-Emerging Diseases and Animal Health Emergencies, established in 2021 to prevent, detect, and respond to animal health emergencies through collective responsibility for animal health security;
- the ASEAN Centre for Public Health Emergencies and Emerging Diseases, organised in 2022 to prepare for, prevent, detect, and respond to public health emergencies and emerging diseases;
- the ASEAN Strategy for Preventing Transmission of Zoonotic Diseases from Wildlife Trade, developed in 2022 to prevent the transmission of zoonotic diseases from wildlife to humans and reduce the demand for illegal wildlife products;
- and the ASEAN One Health Network and Joint Plan of Action, set up on 19 June 2024 to conduct Webinar Series and Policy Briefs on Public Health Emergency based on the priority topics focussing on One Health approach.

In recent years, artificial intelligence (AI) has emerged as a transformative tool in the fight against diseases. For example, AI-based recurrent neural networks and artificial neural network models have shown satisfactory accuracy in predicting the prevalence profile [39] and cumulative incidence of COVID-19 in the USA [40], respectively. Exploratory data analysis and artificial neural network have increased the accuracy of predicting the occurrence of leptospirosis in Malaysia [41]. In the context of One Health, AI could offer novel prospects for understanding, predicting, and mitigating the impact of zoonotic diseases, particularly those caused by bacteria that have acquired antimicrobial resistance genes. By leveraging advanced algorithms and machine learning models, it can process large and complex data sets from various sources, including environmental factors and genetic sequences. This capability allows researchers and public health authorities to make well-informed decisions and implement proactive measures more effectively [42].

Despite the diverse resources, capabilities, and challenges, Southeast Asia has demonstrated a strong commitment in implementing the One Health strategies to combat AMR. However, evaluating the outcomes of these strategies is crucial to identifying effective practices and address shortcomings in the member countries. A systematic assessment could thus help the region identify what works effectively, highlight areas for improvement, and strengthen existing efforts to enhance human, animal, and environmental health. As with other countries and regions worldwide, Southeast Asia also faces the challenge of financial sustainability in the effective implementation of One Health. Regional collaboration on funding mechanisms to reduce the financial burden of individual countries and financial planning to optimise allocation for health, agriculture, and environment sectors are urgently needed in enhancing the One Health strategies across Southeast Asia. In addition, collaboration among governments, health care providers, pharmaceutical companies, and the public to spur innovation, facilitate knowledge sharing and mobilise human resources can further drive progress in combating the AMR crisis.

## References

[1] World Organization for Animal Health (OIE). OIE list of antimicrobial agents of veterinary importance. June 2021. Available: https://www.woah.org/app/uploads/2021/06/a-oie-list-antimicrobials-june2021.pdf. Accessed: 31 August 2024.

[2] Cabello FC Godfrey HP Tomova A Ivanova L Dolz H Millanao A Antimicrobial use in aquaculture re-examined: its relevance to antimicrobial resistance and animal and human health. Environ Microbiol. 2013;15:1917–42. 10.1111/1462-2920.12134 23711078

[3] Jeamsripong S Thaotumpitak V Anuntawirun S Roongrojmongkhon N Atwill ER Hinthong W Molecular epidemiology of antimicrobial resistance and virulence profiles of Escherichia coli, Salmonella spp., and Vibrio spp. isolated from coastal seawater for aquaculture. Antibiotics (Basel). 2022;11:1688. 10.3390/antibiotics11121688 36551345 PMC9774326

[4] Nhinh DT Le DV Van KV Huong Giang NT Dang LT Hoai TD Prevalence, virulence gene distribution and alarming the multidrug resistance of Aeromonas hydrophila associated with disease outbreaks in freshwater aquaculture. Antibiotics (Basel). 2021;10:532. 10.3390/antibiotics10050532 34064504 PMC8147934

[5] Ministry of Health Malaysia, Ministry of Agriculture and Food Security Malaysia. World Malaysian Action Plan on Antimicrobial Resistance (MyAP-AMR) 2022–2026. Kuala Lumpur, Malaysia: Ministry of Agriculture and Food Security, Ministry of Health Malaysia; 2024. Available: https://www.who.int/publications/m/item/malaysia–malaysian-second-action-plan-on-antimicrobial-resistance-(myap-amr). Accessed: 31 August 2024.

[6] Ministry of Public Health Thailand, Ministry of Agriculture and Cooperatives Thailand. National Strategic Plan on Antimicrobial Resistance (2017–2021) At a Glance. https://drug.fda.moph.go.th/media.php?id=502791128794406912&name=05.pdf. Accessed: 31 August 2024.

[7] Ministry of Health Vietnam. National action plan on combating drug resistance in the period from 2013–2020. Hanoi, Vietnam: Ministry of Health Vietnam; 2013. Available: https://cdn.who.int/media/docs/default-source/antimicrobial-resistance/amr-spc-npm/nap-library/vietnam-national-action-plan-on-combatting-drug-resistance-in-the-period-from-2013—2020.pdf?sfvrsn=a7c37ab7_1&download=true. Accessed: 31 August 2024.

[8] Gach MW Lazarus G Simadibrata DM Sinto R Saharman YR Antimicrobial resistance among common bacterial pathogens in Indonesia: a systematic review. Lancet Reg Health Southeast Asia. 2024;26:100414. . 10.1016/j.lansea.2024.100414 38778837 PMC11109028

[9] Chua AQ Kwa AL Tan TY Legido-Quigley H Hsu LY Ten-year narrative review on antimicrobial resistance in Singapore. Singapore Med J. 2019;60:387–96. 10.11622/smedj.2019088 31482178 PMC6717780

[10] World Health Organization. Cumulative number of confirmed human cases of avian influenza A(H5N1) reported to WHO, 2003–2023. 5 January 2023. Available: https://www.who.int/publications/m/item/cumulative-number-of-confirmed-human-cases-for-avian-influenza-a(h5n1)-reported-to-who-2003-2022-5-jan-2023. Accessed: 31 August 2024.

[11] World Organization for Animal Health. Self-declaration on the recovery of freedom from highly pathogenic avian influenza by Malaysia. 17 December 2018. Available: https://www.woah.org/fileadmin/Home/eng/Animal_Health_in_the_World/docs/pdf/Self-declarations/2019_01_Malaysia_HPAI.pdf. Accessed: 31 August 2024.

[12] World Health Organization. Disease outbreak news: Avian influenza – Vietnam. 2 April 2024. Available: https://www.who.int/emergencies/disease-outbreak-news/item/2024-DON511. Accessed: 31 August 2024.

[13] Tan J. No human cases of bird flu in Singapore but public urged to stay vigilant: Ministry of Health. The Straitstime. 21 June 2024. Available: https://www.straitstimes.com/singapore/no-human-cases-of-bird-flu-here-but-singapore-needs-to-be-vigilant-moh Accessed: 31 August 2024.

[14] World Health OrganizationRabies. 5 June 2024. Available: https://www.who.int/news-room/fact-sheets/detail/rabies. Accessed: 31 August 2024.

[15] Bernama. Another new rabies death recorded in Sarawak. The Sun. 11 June 2024. Available: https://thesun.my/local-news/another-new-rabies-death-recorded-in-sarawak-GA12560060. Accessed: 31 August 2024.

[16] World Health Organization. Rabies outbreak response: Accelerating a One Health approach in East Nusa Tenggara. 18 October 2023. Available: https://www.who.int/indonesia/news/detail/18-10-2023-rabies-outbreak-response–accelerating-a-one-health-approach-in-east-nusa-tenggara. Accessed: 31 August 2024*.*

[17] Thanapongtharm W Suwanpakdee S Chumkaeo A Gilbert M Wiratsudakul A Current characteristics of animal rabies cases in Thailand and relevant risk factors identified by a spatial modeling approach. PLoS Negl Trop Dis. 2021;15:e0009980. 10.1371/journal.pntd.0009980 34851953 PMC8668119

[18] Newey S. Vietnam’s ‘dramatic’ rabies surge kills 29 people in four months. The Telegraph. 12 April 2024. Available: https://www.telegraph.co.uk/global-health/science-and-disease/vietnams-rabies-surge-kills-29-people-since-january/ Accessed: 31 August 2024.

[19] Center for Disease Control and Prevention. Rabies status: assessment by country. 31 May 2024. Available: https://www.cdc.gov/rabies/country-risk/index.html. Accessed: 31 August 2024.

[20] Neela VK Philip N Sekawi Z Leptospirosis: Malaysia Leptospirosis Research network experience. Int J Infect Dis. 2021;101:529–46.

[21] World Health Organization. Leptospirosis prevention and control in Indonesia. 24 August 2020. Available: https://www.who.int/indonesia/news/detail/24-08-2020-leptospirosis-prevention-and-control-in-indonesia. *Accessed: 31 August 2024**.*

[22] Limothai U Tachaboon S Dinhuzen J Singh J Jirawannaporn S Seroprevalence of leptospirosis among blood donors in an endemic area. Sci Rep. 2023;13:12336. 10.1038/s41598-023-39461-3 37524788 PMC10390486

[23] Burnham JP Climate change and antibiotic resistance: a deadly combination. Ther Adv Infect Dis. 2021;8:2049936121991374. . 10.1177/2049936121991374 33643652 PMC7890742

[24] Arias-Andres M Klümper U Rojas-Jimenez K Grossart HP Microplastic pollution increases gene exchange in aquatic ecosystems. Environ Pollut. 2018;237:253–61. 10.1016/j.envpol.2018.02.058 29494919

[25] World Health Organization. Global action plan on antimicrobial resistance. Geneva, Switzerland: World Health Organization; 2016. Avaiable: https://www.who.int/publications/i/item/9789241509763. Accessed: 31 August 2024.

[26] Tan HY Overview of Singapore’s One Health Strategy. Zoonotic Dis. 2025;5:2. 10.3390/zoonoticdis5010002

[27] Government of Singapore. Our Story: Learn More About PREPARE’s Aims and Objectives, and Role in Singapore’s Pandemic Preparedness. 22 October 2024. Available: https://www.prepare.gov.sg/about-us/our-story/. Accessed: 13 Mar 2025.

[28] Sumpradit N Wongkongkathep S Malathum K Janejai N Paveenkittiporn W Yingyong T Thailand’s national strategic plan on antimicrobial resistance: progress and challenges. Bull World Health Organ. 2021;99:661–73. 10.2471/BLT.20.280644 34475603 PMC8381094

[29] Boonyasiri A Tangkoskul T Seenama C Saiyarin J Tiengrim S Thamlikitkul V Prevalence of antibiotic-resistant bacteria in healthy adults, foods, food animals, and the environment in selected areas in Thailand. Pathog Glob Health. 2014;108:235–45. 10.1179/2047773214Y.0000000148 25146935 PMC4153825

[30] Food and Agriculture Organization. SIZE Nasional: Harnessing Technology for Effective Control of Infectious Diseases. 23 January 2024. Available: https://www.fao.org/indonesia/news/detail/SIZE-Nasional-Harnessing-Technology-for-Effective-Control-of-Infectious-Diseases/en. Accessed: 13 Mar 2025.

[31] Coyne L Arief R Benigno C Giang VN Huong LQ Jeamsripong S Characterizing antimicrobial use in the livestock sector in three South East Asian countries (Indonesia, Thailand, and Vietnam). Antibiotics (Basel). 2019;8:33. 10.3390/antibiotics8010033 30934638 PMC6466601

[32] Mariappan V Vellasamy KM Mohamad NA Subramaniam S Vadivelu J One Health approaches contribute towards antimicrobial resistance: Malaysian perspective. Front Microbiol. 2021;12:718774. 10.3389/fmicb.2021.718774 34759896 PMC8573254

[33] Sommanustweechai A Tangcharoensathien V Malathum K Sumpradit N Kiatying-Angsulee N Janejai N Implementing national strategies on antimicrobial resistance in Thailand: potential challenges and solutions. Public Health. 2018;157:142–6. 10.1016/j.puhe.2018.01.005 29524812

[34] Lee TH Lye DC Chung DR Thamlikitkul V Lu M Wong AT Antimicrobial stewardship capacity and manpower needs in the Asia Pacific. J Glob Antimicrob Resist. 2021;24:387–94. 10.1016/j.jgar.2021.01.013 33548495

[35] Mitchell MEV Alders R Unger F Nyuyen-Viet H Le TTH Toribio JA The challenges of investigating antimicrobial resistance in Vietnam - what benefits does a One Health approach offer the animal and human health sectors? BMC Public Health. 2020;20:213. 10.1186/s12889-020-8319-3 32046713 PMC7014660

[36] Pan DS Huang JH Lee MH Yu Y Chen MI Goh EH Knowledge, attitudes and practices towards antibiotic use in upper respiratory tract infections among patients seeking primary health care in Singapore. BMC Fam Pract. 2016;17:148. 10.1186/s12875-016-0547-3 27809770 PMC5094024

[37] Goh DL. ASEAN One Health Efforts: Tackling the Intersections of Climate Change and Health. 5 July 2024. Available: https://rsis.edu.sg/wp-content/uploads/2024/07/CO24089.pdf. Accessed: 31 August 2024.

[38] Lajaunie C, Morand S. Southeast Asia at the heart of the implementation of the One Health approach. SSRN [preprint]. 15 September 2023. Available: https://ssrn.com/abstract=4695021*.* Accessed: 13 March 2025.

[39] Kolozsvári LR Bérczes T Hajdu A Gesztelyi R Tiba A Varga I Predicting the epidemic curve of the coronavirus (SARS-CoV-2) disease (COVID-19) using artificial intelligence: an application on the first and second waves. Inform Med Unlocked. 2021;25:100691. 10.1016/j.imu.2021.100691 34395821 PMC8349399

[40] Mollalo A Rivera KM Vahedi B Artificial neural network modeling of novel coronavirus (COVID-19) incidence rates across the continental United States. Int J Environ Res Public Health. 2020;17:4204. 10.3390/ijerph17124204 32545581 PMC7344609

[41] Rahmat F Zulkafli Z Ishak AJ Mohd Noor SB Yahaya H Masrani A Exploratory data analysis and artificial neural network for prediction of leptospirosis occurrence in Seremban, Malaysia Based Meteorological Data. Front Earth Sci. 2020;8:377. 10.3389/feart.2020.00377

[42] Guo W Lv C Guo M Zhao Q Yin X Zhang L Innovative applications of artificial intelligence in zoonotic disease management. Sci One Health. 2023;2:100045. 10.1016/j.soh.2023.100045 39077042 PMC11262289

